# Genomic Analysis of *Sphingopyxis* sp. USTB-05 for Biodegrading Cyanobacterial Hepatotoxins

**DOI:** 10.3390/toxins14050333

**Published:** 2022-05-09

**Authors:** Chao Liu, Qianqian Xu, Zhenzhen Zhao, Haiyang Zhang, Xiaolu Liu, Chunhua Yin, Yang Liu, Hai Yan

**Affiliations:** School of Chemistry and Biological Engineering, University of Science and Technology Beijing, Beijing 100083, China; b20190371@xs.ustb.edu.cn (C.L.); qianqianxu@ustb.edu.cn (Q.X.); b20180362@xs.ustb.edu.cn (Z.Z.); zhanghy@ustb.edu.cn (H.Z.); xiaoluliu@ustb.edu.cn (X.L.); chyin@sina.com (C.Y.); liuyang@ustb.edu.cn (Y.L.)

**Keywords:** *Sphingopyxis*, cyanobacterial hepatotoxins, bacterial biodegradation, genome analysis, phenylacetic acid

## Abstract

*Sphingopyxis* sp. USTB-05, which we previously identified and examined, is a well-known bacterial strain for biodegrading cyanobacterial hepatotoxins of both nodularins (NODs) and microcystins (MCs). Although the pathways for biodegrading the different types of [D-Asp^1^] NOD, MC-YR, MC-LR and MC-RR by *Sphingopyxis* sp. USTB-05 were suggested, and several biodegradation genes were successfully cloned and expressed, the comprehensive genomic analysis of *Sphingopyxis* sp. USTB-05 was not reported. Here, based on second and third generation sequencing technology, we analyzed the whole genome of *Sphingopyxis* sp. USTB-05, which is 4,679,489 bp and contains 4,312 protein coding genes. There are 88 protein-coding genes related to the NODs and MCs biodegradation, of which 16 genes (*bioA*, *hmgL*, *hypdh*, *speE*, *nspC*, *phy*, *spuC*, *murD*, *glsA*, *ansA*, *ocd*, *crnA*, *ald*, *gdhA*, *murC* and *murI*) are unique. These genes for the transformation of phenylacetic acid CoA (PA-CoA) to CO_2_ were also found in *Sphingopyxis* sp. USTB-05. This study expands the understanding of the pathway for complete biodegradation of cyanobacterial hepatotoxins by *Sphingopyxis* sp. USTB-05.

## 1. Introduction

With the rapid development of the world’s agricultural and industrial sectors, a large amount of wastewater and domestic sewage containing nitrogen and phosphorus are discharged into water bodies, resulting in increasing natural water eutrophication. Record-breaking harmful algal blooms and other severe impacts are becoming increasingly frequent [1]. Cyanobacterial hepatotoxins including microcystins (MCs) and nodularins (NODs) are derived from cyanobacteria and are highly toxic, causing a risk to humans and aquatic animals. At least 279 variant structures of MCs have been reported [2]. NODs were also identified to have 12 variant structures [3]. Microcystin-LR (MC-LR), MC-RR, MC-YR, and [D-Asp^1^] NOD have been found and studied. The World Health Organization (WHO) prescribed that the concentration of MC-LR in our drinking water should not be higher than 1.0 μg/L [4]. The lethal dose concentration of nodularins caused by poisoning episodes in certain places is around 50 μg/kg [5,6].

Biodegradation is an efficient and environmentally friendly method to eliminate hepatotoxins. Since *Sphingomonas* ACM-3962 was first reported as a biodegradable MC-LR bacterium in 1994 [7], a variety of bacterial strains for biodegrading MCs from different ecosystems have been found. The majority of strains have been identified as *Sphingomonas* and *Sphingopyxis* of the family Sphingomonadaceae [8,9,10,11]. In surface water, *Sphingomonas* sp. ACM-3962 could biodegrade 1 mg/L MC-LR after a delay period of 2-8 days [12]. However, *Sphingomonas* sp. ACM-3962 has been demonstrated to biodegrade MC-LR, but not [D-Asp^1^] NOD [12]. Further studies found that three enzymatic reaction processes at least involve in MCs biodegradation by *Sphingomonas* sp. ACM-3962 [13], as well as gene clusters involved in MCs biodegradation (*mlrD*, *mlrA*, *mlrB* and *mlrC*) [12,14,15]. Currently, more than 29 MCs biodegrading strains have been identified in Sphingomonadaceae, including 23 strains containing *mlr* gene clusters and 6 strains having biodegradation gene clusters other than *mlr* gene clusters [16,17,18]. Although studies have shown that MCs have many biodegradation pathways, the majority of them have yet to be thoroughly defined [19,20,21].

In 2010, a bacterial strain of *Sphingopyxis* sp. USTB-05 (GenBank accession number: EF607053) was successfully isolated from Lake Dianchi in Yunnan Province of China [22,23], and it was capable of biodegrading both MCs and [D-Asp^1^]NOD [22,23,24,25,26,27]. Initial concentrations of 19.5 mg/L MC-YR, 79.5mg/L MC-RR, 43.6mg/L MC-LR and 25.2 mg/L [D-Asp^1^]NOD were completely biodegraded by *Sphingopyxis* sp. USTB-05 within 4 d [23,24,25,27]. Further studies indicated that MC-LR, MC-YR, MC-RR and [D-Asp^1^]NOD could be biodegraded at more rapid rates by crude enzymes of *Sphingopyxis* sp. USTB-05 with initial MC-YR, MC-LR, MC-RR and [D-Asp^1^]NOD concentration of 14.8, 19.5, 28.4, 25.2 mg/L being completely removed within 12 h [22,23,25,27].

Furthermore, the functions of enzymes encoded by the *USTB-05-A*, *USTB-05-B*, and *USTB-05-C* genes were validated in *E. coli* via heterologous expression [28,29,30,31,32,33,34]. The first enzyme encoded by *USTB-05-A* catalyzes the first step in the biodegrading of MC-YR, MC-LR, MC-RR and [D-Asp^1^]NOD, hydrolyzes the cyclic hepatotoxins into linear hepatotoxins as the first product [28,29,30,31,32]. The second enzyme encoded by *USTB-05-B* can transform linear MC-YR, MC-LR and MC-RR to a tetrapeptide by breaking the Ala-Tyr, Ala-Arg bonds [27,28,33]. The third enzyme encoded by *USTB-05-C* can cleave Adda-Glu peptide bonds and convert the tetrapeptide of Adda-Glu-Mdha-Ala to Adda [33].

Although the bacterial strains, enzymes, and genes for biodegrading both MCs and NODs in Sphingomonadaceae have been well studied, research on the genome of bacterial strains for biodegrading hepatotoxins is rarely reported, and a large number of genes encoding enzymes in the biodegradation pathway of hepatotoxins are rarely clarified. To fully comprehend the biodegradation process, it is essential to identify the corresponding genes and enzymes for biodegrading hepatotoxins through genomic data mining. We analyzed the whole genome of *Sphingopyxis* sp. USTB-05, which 88 genes related to the biodegradation of both NODs and MCs, 16 of which are unique (*bioA*, *hmgL*, *hypdh*, *speE*, *nspC*, *phy*, *spuC*, *murD*, *glsA*, *ansA*, *ocd*, *crnA*, *ald*, *gdhA*, *murC*, and *murI*). These genes for the transformation of phenylacetic acid CoA (PA-CoA) to CO_2_ were also found in *Sphingopyxis* sp. USTB-05.

## 2. Results

### 2.1. General Genome Features of Strain USTB-05

**Figure 1:**
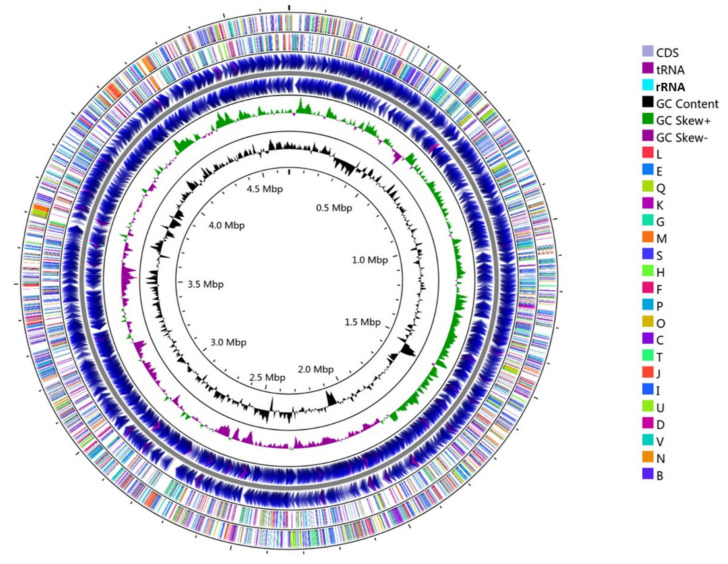
Circular representation of the single chromosome of *Sphingopyxis* sp. USTB-05. From inner to outer ring: circles 1 illustrates position in megabases (black); circles two and three denote GC Content and GC Skew, respectively; circles four and five indicate forward and reverse strand CDS (purple), tRNA (light purple), rRNA (light blue), respectively; circle six is COG analysis of reverse strand CDSs; circle seven is COG analysis of forward strand CDSs. Abbreviations: L, replication, recombination, and repair; E, amino acid transport and metabolism; Q, secondary metabolites biosynthesis, transport and catabolism; K, transcription; M, cell wall, membrane, envelope biogenesis; S, function unknown; H, coenzyme transport and metabolism; F, nucleotide transport and metabolism; P, inorganic ion transport and metabolism; O, posttranslational modification, protein turnover, chaperones; C, energy production and transformation; T, signal transduction mechanisms; J, translation, ribosomal structure, and biogenesis; I, lipid transport and metabolism; U, intracellular trafficking, secretion, and vesicular transport; D, cell cycle control, cell division, chromosome partitioning; V, defense mechanisms; N, cell motility; G, carbohydrate transport and metabolism; B, chromatin structure and dynamics.

The genome of *Sphingopyxis* sp. USTB-05 (4.679 Mb), with an overall GC content of 64%, accounts for 62.39% of the total encoding sequences (Figure 1 and Table 1). Without a CRISPR site, the genome sequences of strain USTB-05 comprises 4312 predicted protein-encoding sequences. Forty-eight tRNAs and one tmRNA were identified (Table 1). The 16S rRNA of strain USTB-05 is one copy gene within a single genome.

### 2.2. Gene Ontology Annotation

**Figure 2:**
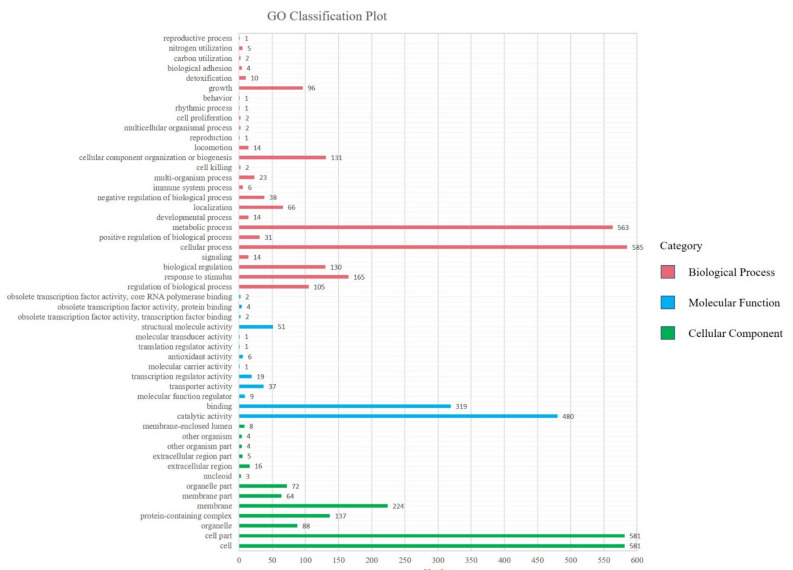
Distributions of second level GO of *Sphingopyxis* sp. USTB-05 genome sequence. The *y*-axis indicates the GO ontology; the *x*-axis represents the number of unigenes in a category.

Gene ontology (GO) is a standardized gene functional classification system that tenders to a dynamic-updated controlled vocabulary. In the GO database, gene functions are categorized as biological processes, cellular components, and molecular functions. The GO analysis indicates that a total of 4,731 GO terms are associated with all unigenes (Figure 2, Supplementary Table S1). According to the secondary classification of the GO terms, all unigenes are sorted into 49 functional groups. Biological process is the main category of GO annotations (2,012, 42.53%) unigenes, followed by cellular component (1,787, 37.77%) and molecular function (932, 19.70%). Most of the biological process categories are represented by the cellular process (29.08%) and metabolic process (27.98%), suggesting that the bacterium has strong metabolic activity. There are also some subcategories including response to stimulus (8.20%), cellular component organization or biogenesis (6.51%), biological regulation (6.46%), regulation of biological process (5.22%), growth (4.77%) and localization (3.28%). Cell (32.51%), cell part (32.51%), membrane (12.53%) and protein containing complex (7.67%) are the cell gene clustering of three main components. The catalytic activity (51.50%) and binding (34.23%) represent most of the molecular function category, forecasting that the bacterium has a high degree of molecular catalysis (Figure 2).

### 2.3. Cluster of Orthologous Groups Classification

**Figure 3:**
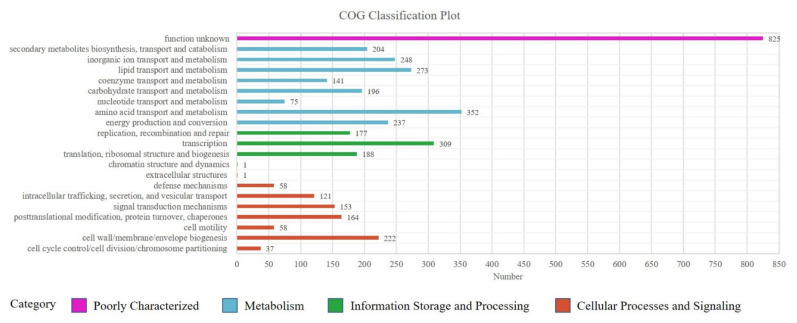
COG functional classification of *Sphingopyxis* sp. USTB-05. The columns represent the number of unigenes in each subcategory.

The Cluster of Orthologous Groups (COG) is a database used to classify gene products based on their homology. Unigenes *Sphingopyxis* sp. USTB-05 are annotated in the COG database is 62.39%. A total of 4040 classified unigenes are divided into 25 functional categories. The four main groups of amino acid transport and metabolism (352, 8.71%), transcription (309, 7.65%), lipid transport and metabolism (273, 6.76%), and function unknown (825, 20.42%) are the most prevalent. The biodegradation of Adda is completed by carbohydrate transport and metabolism (4.85%), this is also a key category to consider. In addition, the biodegradation of cyanobacterial hepatotoxins is dependent on various biological enzymes during cellular processes and signaling (20.15%). Thus, posttranslational modification, protein turnover, chaperones (4.06%) are also considered an important functional group (Figure 3, Supplementary Table S2).

### 2.4. Genes and Gene Clusters Associated with Hepatotoxins Biodegradation

The whole genome of *Sphingopyxis* sp. USTB-05 contains many genes related to nutrient absorption, including *ssuA*, *modA*, *potD*, etc. They are all classified as the ABC transporters pathway. *Sphingopyxis* sp. USTB-05 primarily has five metabolic processes (alanine, aspartate, and glutamate metabolism; arginine and proline metabolism; D-glutamine and D-glutamate metabolism; degradation of aromatic compounds; and valine, leucine, and isoleucine biosynthesis), all of which are related to the biodegradation of hepatotoxin (Table 2).

**Figure 4:**
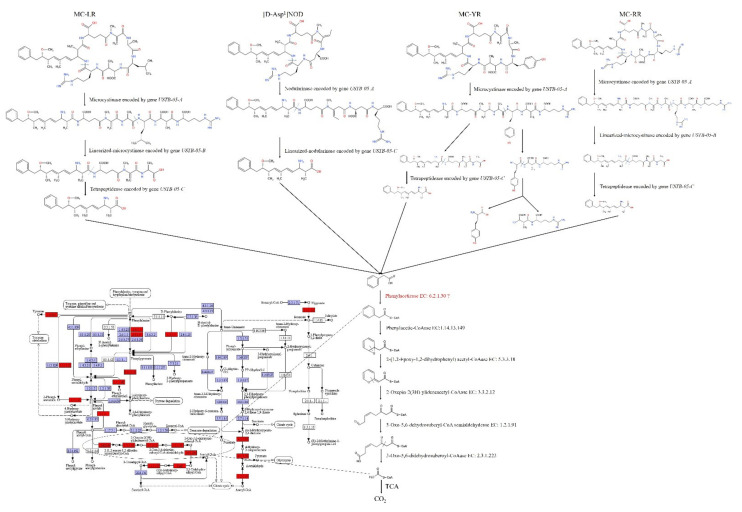
The metabolism pathway for biodegrading cyanobacterial hepatotoxins by *Sphingopyxis* sp. USTB-05. In this purposed metabolism pathway, MC-LR, MC-YR, MC-RR and [D-Asp^1^]NOD are enzymatically hydrolyzed by glutamate protease USTB-05-A to produce linearized MC-LR, MC-YR, MC-RR and [D-Asp^1^]NOD, which are further biodegraded by USTB-05-B or USTB-05-C to tetrapeptide or Adda. Tetrapeptide is disassimilated to Adda by tetrapeptidease. After, Adda is biodegraded into PA, which is further biodegraded by the unannotated enzymes to PA-CoA. Then PA-CoA is biodegraded to acetyl-CoA by the corresponding Phenylacetic-CoAase; 2-(1,2-Epoxy-1,2-dihydrophenyl) acetyl-CoAase; 2-Oxepin-2(3H)-ylideneacetyl-CoAase; 3-Oxo-5,6-dehydrosuberyl-CoA semialdehydease and 3-Oxo-5,6-didehydrosuberoyl-CoAase. Ultimately, acetyl-CoA is completely converted to CO_2_ via the TCA cycle. The dashed line indexes the sites of bond-breakage. The red boxes represent that these genes are found in *Sphingopyxis* sp. USTB-05 genome. The purple or white boxes represent that these genes are not noted in *Sphingopyxis* sp. USTB-05 genome.

In the KEGG pathway database, we performed comparative analysis of these functional genes from *Sphingopyxis* sp. USTB-05 and *Sphingomonas morindae* sp. NBD5 (Figure S2). These genes *potD*, *potC*, *potB*, *porG*, *malK*, *msmX*, *smoK*, *thuK,* and *lapB* in the metabolic pathways of ABC transporters are peculiar to *Sphingopyxis* sp. USTB-05. *crnA*, *phy*, *ocd*, *hypdh*, *spuC*, *nspC,* and *speE* are specific to *Sphingopyxis* sp. USTB-05 in the metabolic pathways of arginine and proline. *ald*, *ansA,* and *gdhA* are unique to *Sphingopyxis* sp. USTB-05 in the metabolic pathways of alanine, aspartate and glutamate. *murI*, *murC,* and *murD* are characteristic to *Sphingopyxis* sp. USTB-05 in the metabolic pathways of D-glutamine and D-glutamate. *glsA*, *bioA,* and *hmgL* in the biodegradation pathway of valine, leucine and isoleucine may be involved in the biodegradation of cyanobacterial hepatotoxins. Interestingly, *Sphingopyxis* sp. USTB-05 were predicted to contain a complete set of genes involved in phenylacetate biodegradation, in addition to the gene encoding AMP-forming phenylacetyl-CoA ligase (PA-CoA ligase; EC: 6.2.1.30) (Figure 4). *paaR*, *paaE*, *paaC*, *paaB*, *paaA*, *paaG,* and *paaN* of phenylacetate biodegradation are important in *Sphingopyxis* sp. USTB-05.

## 3. Discussion

Biodegradation is an efficient way to remove cyanobacterial hepatotoxins from water bodies. 16S rRNA-oriented phylogeny is used to evaluate the taxonomic placement of bacteria [10]. In 2010, 16S rDNA sequence analysis showed that the *Sphingopyxis* sp. USTB-05 was most similar to the reference strain *Sphingopyxis* sp. C-1 [22]. However, evolutionary relations among the *Sphingopyxis* genus of MC-biodegrading bacteria were re-evaluated. *Sphingopyxis* sp. USTB-05 is the most similar the reference strain *Sphingopyxis chilensis* S37, having a similarity of 99.29. Recent studies demonstrate that genome-wide phylogeny can improve the phylogenetic accuracy and preferably delimit the species borderlines [35]. *Sphingopyxis* sp. USTB-05 whole-genome sequencing enriches the whole genome data of cyanobacterial hepatotoxins biodegrading bacteria. For instance, the taxonomic study of *Sphingosinicella* sp. B9, *Sphingopyxis* sp. C-1 and *Novosphingobium* sp. MD-1 suggests that they are phylogenetically different from previously described species [36,37,38,39].

Certain amino acid metabolic processes may be involved in the biodegradation of [D-Asp^1^]NOD, MC-LR, MC-YR, and MC-RR. Due to cyanobacterial hepatotoxins [D-Asp^1^]NOD, MC-LR, MC-YR, and MC-RR are a class of monocyclic pentapeptide and heptapeptide compounds. According to the cyanobacterial hepatotoxins general chemical molecular structures, contain amino acids such as the following: D-isoleucine, D-alanine, D-glutamic acid, D-erythro-β-methylaspartic acid, variable L-amino acid, L-arginine, N-dehydrogenation alanine, Adda. The variable L-amino acids are arginine and leucine for the congener MC-LR. Adda is a remarkable C20 β-amino acid: (2S, 3S, 8S, 9S) 3-amino-9-methoxy-2, 6, 8-trimethyl-10-phenyldeca-4(E), 6(E)-dienoic acid [40]. MC-LR and MC-LA biodegrading bacteria have been identified in a variety of genera, including *Brevibacterium* sp., *Rhodococcus* sp. and *Arthrobacter* sp. [41,42,43], but reports of highly biodegradable hepatotoxins MCs in the Sphingomonadaceae are increasing [14]. *Sphingopyxis* sp. USTB-05 gene clusters for [D-Asp^1^]NOD, MC-LR, MC-YR and MC-RR biodegradation were identified, including *USTB-05-A*, *USTB-05-B,* and *USTB-05-C*. Molecular studies find that *Sphingopyxis* sp. USTB-05 contains *USTB-05-A*, *USTB-05-B* and *USTB-05-C* genes, which are highly homologous to *mlr A*, *mlr B,* and *mlr C*, respectively [28]. The first enzyme encoded by *USTB-05-A* stimulates the first and most critical step in the biodegrading of [D-Asp^1^]NOD, MC-LR, MC-YR, and MC-RR, including the stimulation of cyclic hepatotoxins to linear hepatotoxins as the first product [27,29,30,31,32]. The second enzyme encoded by *USTB-05-B* converts linear [D-Asp^1^]NOD, MC-LR, MC-YR, and MC-RR to tetrapeptide by cutting off the Ala-Arg, Ala-Tyr bonds [27,28,31,33]. The third enzyme encoded by *USTB-05-C* cleaves the Adda-Glu peptide bond, transforming it to Adda-Glu-Mdha-Ala into Adda [33] (Figure 4).

Hashimoto et al. [44] noted that genes referred to the biodegradation of MC-LR comprised a lot more than these four genes. However, there are few genomic data on strains that biodegrade cyanobacterial hepatotoxins, limiting further research on the biodegradation mechanism. Via the KEGG database metabolic pathway annotation, the following genes may be referred to the biodegradation processes of [D-Asp^1^]NOD, MC-LR, MC-YR, and MC-RR by *Sphingopyxis* sp. USTB-05. These genes *gdhA*, *ansA,* and *ald* in the metabolic pathways of glutamate, alanine and aspartate are referred to the biodegradation of D-glutamate, D-alanine and D-erythro-β-methylaspartate. The involving biodegradation genes of L-arginine are *speE*, *crnA*, *hypdh*, *nspC*, *ocd*, *spuC,* and *phy* in the metabolic pathway of proline and arginine. *murC*, *murD,* and *murI* take part in the biodegradation of D-glutamate. *glsA*, *bioA* and *hmgL* participate in the biodegradation of D-isoleucine (Table 2).

Adda is the detoxification end-product produced by the final enzymatic reaction. *Sphingopyxis* sp. USTB-05 was forecasted to possess a full set of genes taken part in phenylacetate biodegradation, in addition to the gene encoding AMP-forming phenylacetyl-CoA ligase (PA-CoA ligase) (Figure 4). Recently, genes and transposable elements associated with phenylacetate biodegradation have been identified near the *mlr* gene cluster [36,45]. A previous report [36,37] demonstrated the identification of a set of genes involved in the phenylacetate biodegradation in *Sphingopyxis* sp. C-1. However, the gene encoding phenylacetyl-CoA ligase was absent. *Sphingopyxis* sp. YF1, which leans on the *mlr* biodegradable metabolic pathway, can also biodegrade Adda by the phenylacetic acid metabolism pathway [46].

## 4. Conclusions

The whole genome of *Sphingopyxis* sp. USTB-05 consists of a circular chromosome of 4,679,489 bp with 4312 protein-coding genes including 88 genes related to the biodegradation of both NODs and MCs, of which 16 genes (*bioA*, *hmgL*, *hypdh*, *speE*, *nspC*, *phy*, *spuC*, *murD*, *glsA*, *ansA*, *ocd*, *crnA*, *ald*, *gdhA*, *murC,* and *murI*) are unique. These genes for the transformation of phenylacetic acid CoA (PA-CoA) to CO_2_ were also found in *Sphingopyxis* sp. USTB-05. This study expands the understanding of the pathway for complete biodegradation of cyanobacterial hepatotoxins by *Sphingopyxis* sp. USTB-05.

## 5. Materials and Methods

### 5.1. Bacterial Strains

*Sphingopyxis* sp. USTB-05 was isolated and identified from the sediment of Dianchi Lake in Kunming, Yunnan, China [22].

### 5.2. DNA Extraction and Sequencing

*Sphingopyxis* sp. USTB-05 was initially incubated on the original solid isolation media at 30 °C for 48 h. A single colony was selected and cultivated in the culture medium of previous report [22]. The genomic DNA was extracted using the Rapid Bacterial Genomic DNA Isolation Kit (CoWin Biosciences, Taizhou, Jiangsu, China) according to the manufacturer’s instructions. NanoDrop (Thermo Fisher Scientific, Waltham, MA, USA) analysis and gel electrophoresis were used to determine the purity and concentration of the DNA samples. A small fragment second-generation genomic library with a size of 350 bp was constructed using the NEBNext^®^ Ultra™ II DNA kit. The genome was sequenced by using Illumina X10 platform (Madison, WI, USA) [47]. The third-generation genomic library was structured by the standard protocol of Oxford Nanopore Technologies (ONT, Oxford, UK).

### 5.3. Genome Assembly and Quality Control

Prior to genome assembly, the qualities of the next-generation sequencing reads were optimized by fastp software v0.23.2 before assembly. The sequences with a quality value of Q < 25 and containing linker fragments were deleted. The first fastq formatted data for nanopore sequencing was gathered using FAST5 files included in the MinKNOW software v4.0.4 package. For genome assembly, a total of 11 Mb Nanopore long reads with an N50 length of 8 kb were produced (Figure S1). Spades software (combined with its development process) was used for hybrid assembly, while Pilon software v1.5 was used to correct the assembly results.

### 5.4. Genome Annotation

The online NMPDR-rust server was used to forecast the gene and coding sequence (CDs). All unigenes were functionally annotated using the Pfam and Swiss-Prot databases. Circos calling a visualization tool was effective in evidencing variation in the genome’s structure. The annotation of eggNOG of protein-coding genes was completed by blast software v2.9.0 [48]. The GO annotation of protein-coding genes were annotated using the Pfam and SwissProt databases. Kobas 3.0 software was used to document KO pathway annotations of protein-coding genes. In addition, the CRISPRFinder software v4.2.19 was used to forecast the clustered regularly interspaced short palindromic repeats (CRISPR) structure of the *Sphingopyxis* sp. USTB-05 genome [49]. The coding sequences of the genome were arranged for using MUMmer version 4.0+ and analyzed in combination with the results of the genome annotation [50].

### 5.5. Nucleotide Sequence Accession Number

The sequence data were submitted to NCBI Sequence Read Archive (https://www.ncbi.nlm.nih.gov/sra/ (accessed on 6 March 2022)) with accession numbers CP084712, CP084930-CP084933. The sequence data will be released on 31 October 2023.

## Figures and Tables

**Table 1:** Genome characteristics of *Sphingopyxis* sp. USTB-05.

Category	*Sphingopyxis* sp. USTB-05
bases	4,679,489
tmRNA	1
tRNA	48
CDS	4312
GC(%)	64%
plasmid	0

**Table 2:** Genes related to cyanobacterial hepatotoxin biodegradation in the genome of *Sphingopyxis* sp. USTB-05.

Pathway	Genes
ABC transporters	*ssuA*, *ssuC*, *ssuB*, *modA*, *modB*, *modC*, *potD* *, ***potC***, ***potB***, ***porG***, ***malK***, ***msmX***, ***smoK***, ***thuK***, *mlaD*, *mlaE*, *mlaF*, *pstS*, *pstC*, *pstA*, *pstB*, *lolC*, *lolE*, *lolD*, *ccmC*, *ccmB*, *ccmA*, *lptF*, *lptB*, *lptG*, *ftsX*, *ftsE*, *hlyB*, ***lapB***
Alanine, aspartate and glutamate metabolism	***ald***, *pyrB*, *purA*, *argG*, *purB*, *argH*, *asnB*, ***ansA***, *racD*, *nadB*, *gabD*, *gdhB*, ***gdhA***, *gltB*, *glmS*, *glnA*, *purF*, *cad*
Arginine and proline metabolism	*astB*, *astA*, ***crnA***, *proB*, *putA*, *proC*, ***phy***, *pip*, ***ocd***, ***hypdh***, *amiE*, ***spuC***, ***nspC***, ***speE***
D-glutamate and D-glutamine metabolism	***murI***, ***murD***, ***murC***
Valine, leucine and iso-leucine degradation	*ilvE*, *bkdA1*, *pdhD*, *bkdB*, *ivd*, *paaF*, *pccB*, ***hmgL***, *scoA*, *atoB*, *mmsB*, ***bioA***, *bccA*, *sbm*, *mce*, *aspC*, ***glsA***, *mssA*, *proA*

* The bold fonts indicate that these genes are only present in *Sphingopyxis* sp. USTB-05 genome, but not in *Spingomonas morindae* sp. NBD5.

## Data Availability

Not applicable.

## Supplementary Materials

The following supporting information can be downloaded at: https://www.mdpi.com/2072-6651/14/5/333/s1. Table S1: Gene classification results: Gene Ontology (GO) classifications of genes. It includes the hierarchy of GO and gene number in this GO term; Table S2: Gene classification results: Clusters of Orthologous Group (COG) classifications of genes. It shows the COG class, abbreviation, gene numbers and IDs in the COG term; Figure S1: The length of the three generations of nanopore data is distributed in turn; Figure S2: Comparison of genes related to hepatotoxin biodegradation in the genomes of *Sphingomonas morindae* sp. NBD5 and *Sphingopyxis* sp. USTB-05.
