# Supplementary material for: Genomic Analysis of Sphingopyxis sp. USTB-05 for Biodegrading Cyanobacterial Hepatotoxins
Source: Toxins (Basel). 2022 May 9;14(5):333. doi: 10.3390/toxins14050333 (PMC9144602; doi:10.3390/toxins14050333)
Supplement: Supplementary file 1 [file toxins-14-00333-s001.zip › toxins-1635261 supplementary figure.pdf]

Article

Genomic Analysis of *Sphingopyxis* sp. USTB-05 for Biodegrading Cyanobacterial Hepatotoxins

Chao Liu, Qianqian Xu, Zhenzhen Zhao, Haiyang Zhang, Xiaolu Liu, Chunhua Yin, Yang Liu, and Hai Yan

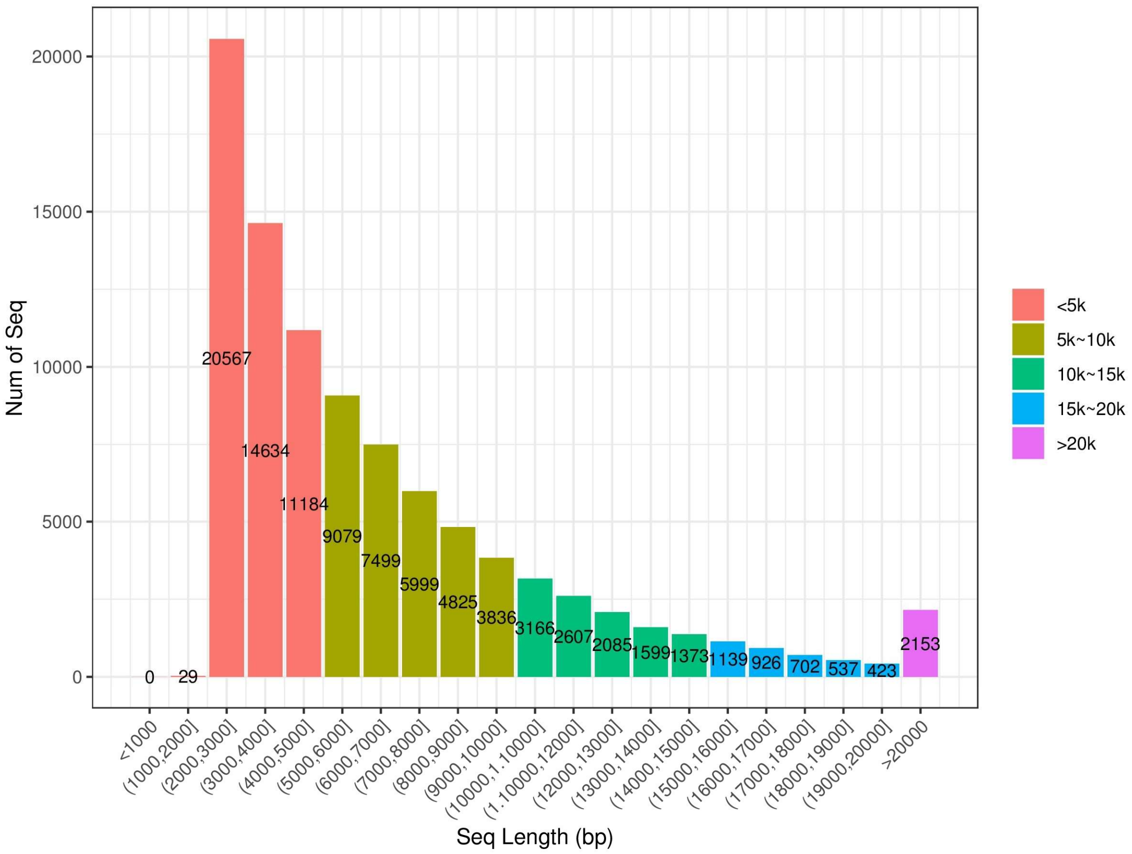

Figure S1. The length of the three generations of nanopore data is distributed in turn.

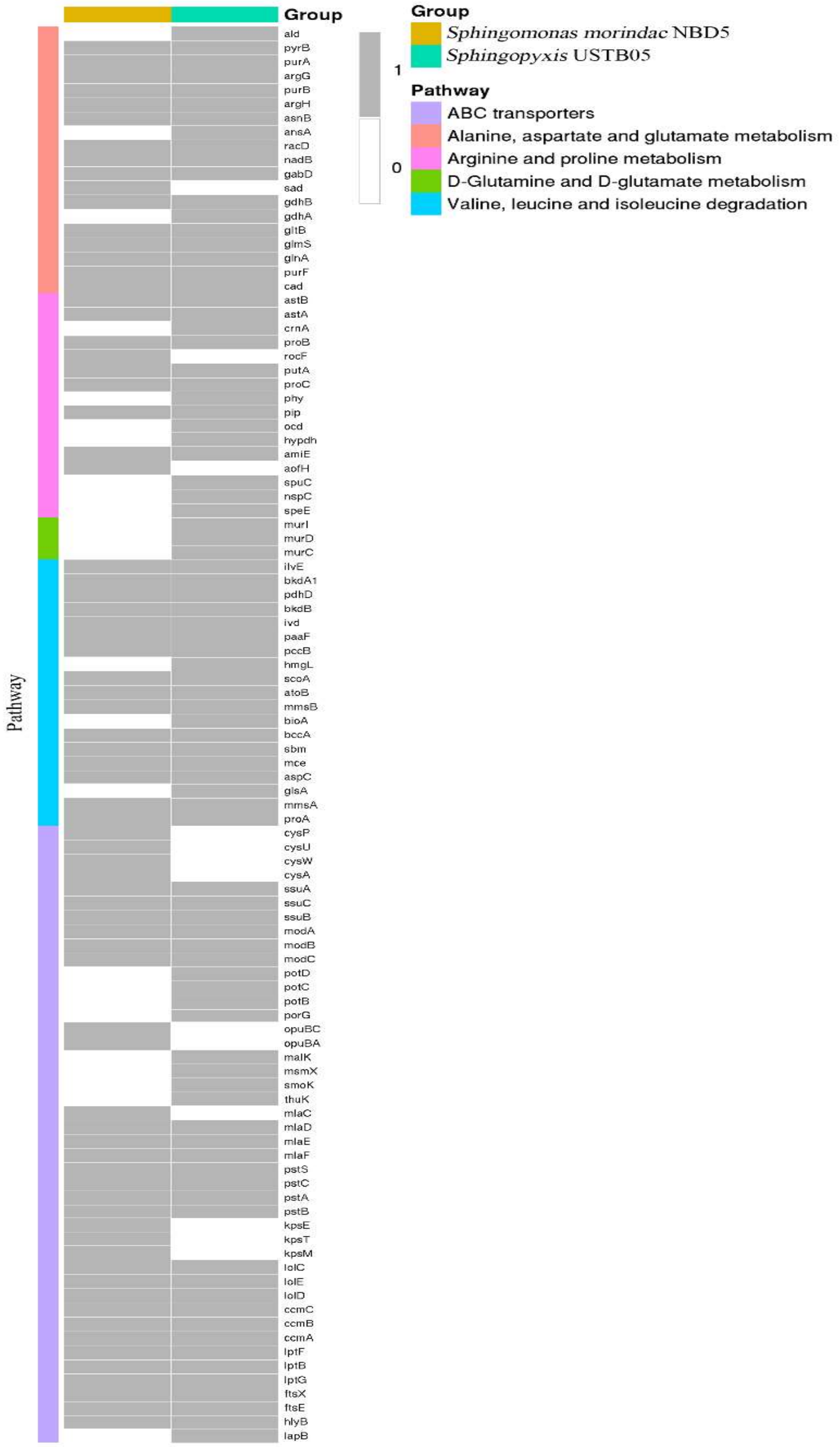

Figure S2. Comparison of genes related to hepatotoxin biodegradation in the genomes of *Sphingomonas morindae* sp. NBD5 and *Sphingopyxis* sp. USTB-05. The gray box in the figure indicate that the gene exists in two strains, and the white indicate that the gene does not exist in two strains.
